# Asymptomatic and symptomatic cardiac toxicity associated with immune checkpoint inhibitors: insights from a Japanese registry

**DOI:** 10.1093/ehjopen/oeag079

**Published:** 2026-05-15

**Authors:** Yudai Tamura, Yuichi Tamura, Hiroshi Ohtsu, Shiro Nakamori, Kazuaki Maruyama, Nao Muraoka, Masaaki Shoji, Kazuko Tajiri, Hirofumi Tomita, Yoshitaka Iso, Koichiro Sugimura, Hirotsugu Yamada, Koichiro Kinugawa, Ryota Morimoto, Hiroshi Akazawa, Kazumasa Harada, Shintaro Nakano, Kenichi Tsujita, Tadahiro Gunji, Toshihiro Tsuruda, Akihiro Nakamura, Michiro Maruyama, Yuichi Miyake, Kunihiko Hatanaka, Hiroaki Kawano, Wakana Sato, Shingo Yano, Kyoko Imanaka-Yoshida, Issei Komuro

**Affiliations:** Pulmonary Hypertension Center, International University of Health and Welfare Mita Hospital, 1-4-3 Mita, Minato-ku, Tokyo 108-8329, Japan; Department of Cardiology, Kanazawa Cardiovascular Hospital, Ha-16 Tanakamachi, Kanazawa, Ishikawa 920-0007, Japan; Pulmonary Hypertension Center, International University of Health and Welfare Mita Hospital, 1-4-3 Mita, Minato-ku, Tokyo 108-8329, Japan; Department of Cardiology, International University of Health and Welfare School of Medicine, 4-3 Kozunomori, Narita, Chiba 286-8686, Japan; Faculty of Health Data Science, Juntendo University, 6-8-1 Hinode, Urayasu, Chiba 279-0013, Japan; Department of Cardiology and Nephrology, Mie University Graduate School of Medicine, 2-174 Edobashi, Tsu, Mie 514-8507,Japan; Department of Pathology and Matrix Biology, Mie University Graduate School of Medicine, 2-174 Edobashi, Tsu, Mie 514-8507, Japan; Division of Cardiology, Shizuoka Cancer Center, 1007 Shimonagakubo, Nagaizumi-cho, Sunto-gun, Shizuoka 411-8777, Japan; Department of Cardiovascular Medicine, National Cancer Center Hospital, 5-1-1 Tsukiji, Chuo-ku, Tokyo 104-0045, Japan; Department of Cardiology, Institute of Medicine, University of Tsukuba, 1-1-1 Tennodai, Tsukuba, Ibaraki 305-8575, Japan; Department of Cardiology and Nephrology, Hirosaki University Graduate School of Medicine, 5 Zaifu-cho, Hirosaki, Aomori 036-8562, Japan; Division of Cardiology, Department of Internal Medicine, Showa Medical University Fujigaoka Hospital, 1-30 Fujigaoka, Aoba-ku, Yokohama, Kanagawa 227-8501, Japan; Department of Cardiology, International University of Health and Welfare Narita Hospital, 4-3 Kozunomori, Narita, Chiba 286-8686,Japan; Department of Community Medicine for Cardiology, Tokushima University Graduate School of Biomedical Sciences, 3-18-15 Kuramoto-cho, Tokushima 770-8503, Japan; The Second Department of Internal Medicine, University of Toyama, 2630 Sugitani, Toyama 930-0194, Japan; Department of Cardiology, Nagoya University Graduate School of Medicine, 65 Tsurumai-cho, Showa-ku, Nagoya, Aichi 466-8550,Japan; Department of Cardiovascular Medicine, Graduate School of Medicine, The University of Tokyo, 7-3-1 Hongo, Bunkyo-ku, Tokyo 113-8655, Japan; Department of Cardiology, Tokyo Metropolitan Institute for Geriatrics and Gerontology, 35-2 Sakae-cho, Itabashi-ku, Tokyo 173-0015,Japan; Department of Cardiology, Saitama Medical University International Medical Center, 1397-1 Yamane, Hidaka, Saitama 350-1298, Japan; Department of Cardiovascular Medicine, Graduate School of Medical Sciences, Kumamoto University, 1-1-1 Honjo, Chuo-ku, Kumamoto 860-8556, Japan; Division of Clinical Oncology/Hematology, Department of Internal Medicine, The Jikei University School of Medicine, 3-25-8 Nishi-Shimbashi, Minato-ku, Tokyo 105-8461, Japan; Department of Hemo-Vascular Advanced Medicine, Faculty of Medicine, University of Miyazaki, 5200 Kihara, Kiyotake-cho, Miyazaki 889-1692, Japan; Department of Cardiology, Iwate Prefectural Central Hospital, 1-4-1 Ueda, Morioka, Iwate 020-0066, Japan; Department of Cardiology, Toyama Prefectural Central Hospital, 2-2-78 Nishinagae, Toyama 930-8550,Japan; Department of Cardiorenal and Cerebrovascular Medicine, Faculty of Medicine Kagawa University, 1750-1 Ikenobe, Miki-cho, Kita-gun, Kagawa 761-0793, Japan; Department of Cardiology, Japanese Red Cross Society Himeji Hospital, 1-12-1 Shimoteno, Himeji, Hyogo 670-8540, Japan; Department of Cardiovascular Medicine, Nagasaki University Graduate School of Biomedical Sciences, 1-7-1 Sakamoto, Nagasaki 852-8501, Japan; Department of Cardiovascular Medicine, Akita University Graduate School of Medicine, 1-1-1 Hondo, Akita 010-8543, Japan; Division of Clinical Oncology/Hematology, Department of Internal Medicine, The Jikei University School of Medicine, 3-25-8 Nishi-Shimbashi, Minato-ku, Tokyo 105-8461, Japan; Department of Cardiology and Nephrology, Mie University Graduate School of Medicine, 2-174 Edobashi, Tsu, Mie 514-8507,Japan; Department of Pathology and Matrix Biology, Mie University Graduate School of Medicine, 2-174 Edobashi, Tsu, Mie 514-8507, Japan; Department of Frontier Cardiovascular Science, Graduate School of Medicine, The University of Tokyo, 7-3-1 Hongo, Bunkyo-ku, Tokyo 113-0033, Japan; International University of Health and Welfare School of Medicine, 4-1-26 Akasaka, Minato-ku, Tokyo 107-8402, Japan

**Keywords:** Immune checkpoint inhibitors, Cardiac complication, Cardiac toxicity, Myocarditis, Registry

## Abstract

**Aims:**

Immune checkpoint inhibitors (ICIs) are a cornerstone of cancer therapy; however, immune-related adverse events, including myocarditis, pose significant challenges. Furthermore, limited data exist on the pathology of mild or asymptomatic cases. This study aimed to characterize the clinical, biomarker, and pathological features of ICI-associated cardiac toxicity, comparing symptomatic and asymptomatic cases within a Japanese multicentre registry.

**Methods and results:**

A nationwide, retrospective registry collected data from 90 patients across 23 hospitals between 2020 and 2022. Patients were classified according to the American Society of Clinical Oncology Clinical Practice Guidelines: Grade 1 (asymptomatic) and Grades 2–4 (symptomatic). Endomyocardial biopsy was performed in 24 patients (*n* = 19 symptomatic, *n* = 5 asymptomatic). Among the enrolled patients (mean age: 68 years; 76.7% male), 41.1% were classified as Grade 1, whereas 58.9% were symptomatic (Grades 2–4). Symptomatic cases exhibited significantly higher troponin I and creatine kinase levels at onset. Pathological analysis revealed more extensive lymphocytic infiltration (CD3+ T cells) in symptomatic cases, particularly with higher CD8+ and CD68+ cell counts. Continuation of ICI therapy was more frequent in the asymptomatic group, and only one patient experienced recurrence. In contrast, all seven myocarditis-related deaths occurred in symptomatic patients.

**Conclusion:**

This study provides one of the largest pathological and biomarker-based comparisons of symptomatic and asymptomatic ICI-associated cardiac toxicity. Our findings suggest that asymptomatic cases may represent a distinct, less aggressive inflammatory phenotype, characterized by lower CD8+ infiltration and limited myocardial damage.

## Graphical Abstract

**Graphical Abstract:**
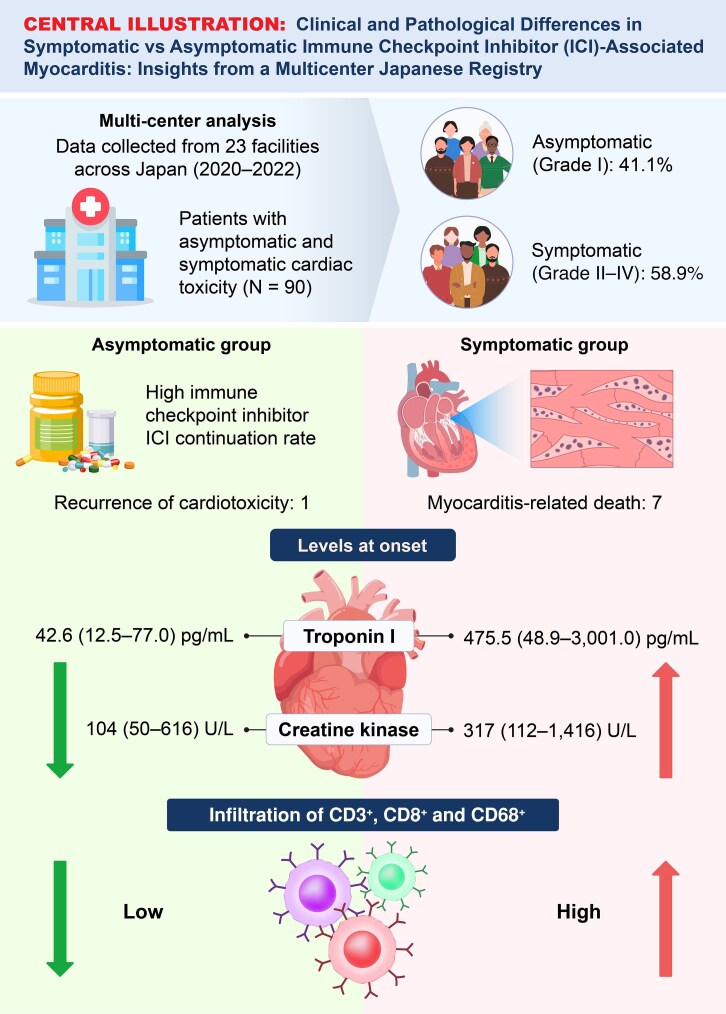

## Introduction

Immune checkpoint inhibitors (ICI) have improved cancer prognosis and revolutionized cancer treatment. Immune checkpoint inhibitors have been established as one of the main therapies across various cancer types and clinical settings. Although ICIs stimulate the immune system to produce anti-tumour effects, they also cause immune-related adverse events (irAEs) in various organs in most patients. Among these, ICI-associated myocarditis is one of the most serious irAEs, with a high mortality rate.^1^ Recently, the mortality rate for ICI-associated myocarditis has declined,^2,3^ partly owing to increased awareness of this irAE.^4^ Additionally, some patients with cardiac test abnormalities, including elevated cardiac troponin and abnormal electrocardiogram, cannot be definitively diagnosed as myocarditis. Moreover, certain patients exhibit no symptoms but have pathological autopsy findings suggestive of myocarditis.^5–8^ Importantly, asymptomatic ICI-associated myocarditis poses a distinct clinical challenge, as the absence of overt symptoms may delay recognition while myocardial inflammation is already present. Failure to identify these cases may lead to progression to severe cardiotoxicity, whereas overdiagnosis may result in unnecessary interruption of life-prolonging cancer therapy. Therefore, improved characterization of asymptomatic cardiac involvement is essential to balance cardiovascular safety and oncological benefit. Currently, few studies have investigated asymptomatic myocarditis and cardiotoxicity-associated ICI therapy. Furthermore, compared to symptomatic ICI-associated myocarditis, the pathological features of patients with asymptomatic cardiotoxicity associated with ICI therapy remain unknown. Recommendations for monitoring cardiotoxicity are given in several guidelines; however, due to the low incidence of ICI-associated myocarditis, the usefulness of detecting cardiotoxicity has not been verified.^9,10^ However, since discontinuing ICI worsens cancer prognosis, it is important to investigate the characteristics of ICI-induced cardiotoxicity, with or without symptoms. Hence, minimizing the unnecessary discontinuation of ICI is a critical goal in cardio-oncology.^11^

In Japan, a multicentre registry has been established to collect data on ICI-associated cardiac toxicity. To the best of our knowledge, this is the first registry study in Japan to investigate ICI-associated cardiotoxicity. Uniquely, this registry couples systematic biomarker surveillance with centralized pathological review, enabling side-by-side evaluation of tissue findings and clinical phenotype.

Building on this registry, this study aimed to clarify the clinical and pathological distinctions between symptomatic and asymptomatic myocardial damage. Our findings are expected to provide critical insights to guide clinical decisions regarding when—and whether—to continue ICI therapy despite potential cardiotoxicity risks.

## Methods

### Study participants

This retrospective study utilized a registry of patients in Japan who were diagnosed with cardiac toxicity while receiving ICI. This registry includes all suspected cases of ICI-associated myocarditis at participating hospitals. This study was a retrospective observational study in which previously treated patients were retrospectively identified and entered into a registry based on existing medical records. The enrolment period concluded in December 2022. Depending on the judgment of each hospital, appropriate combinations of coronary computed tomography, cardiac magnetic resonance imaging, cardiac catheterization, and myocardial biopsy were performed for differential diagnosis. All patients in this registry were included in this study. In this study, cardiac toxicity was classified into four severity grades according to the American Society of Clinical Oncology Clinical Practice Guideline in effect at the start of the registry.^12,13^ Grade 1 was defined as abnormalities in cardiac biomarkers, including electrocardiogram; Grade 2 as abnormalities in screening tests with mild symptoms; Grade 3 as moderate abnormalities in tests or symptoms with mild activity; and Grade 4 as moderate to severe decompensated heart failure requiring intravenous medication, intervention, or posing life-threatening conditions. Symptoms in this study were defined as cardiac symptoms. We classified Grade 1 patients (mild or subclinical changes) as the asymptomatic group, whereas Grade 2–4 patients were classified as the symptomatic group. Subsequently, the two groups were compared to evaluate differences in clinical, biomarker, and pathological features. We excluded patients who did not have sufficient evidence to indicate at least ‘Possible myocarditis,’ based on Bonaca’s diagnostic criteria.^14^ These decisions were made by the physicians treating the patient at each hospital. ASCO grading was used solely to classify symptom severity (Grade 1 vs. Grades 2–4). Diagnostic certainty was adjudicated using Bonaca definitions and cross-checked against ESC 2022 criteria where data permitted.

### Evaluation and definition of clinical variables

We collected basic characteristic data, including age (at the start of ICI administration), sex, type of cancer and ICIs used, concomitant drugs, comorbidities, date of diagnosis for cardiac toxicity, irAE affecting other organs, findings from cardiac test, management of cardiac events, and clinical outcome. The baseline data was defined as the data before the administration of ICI used in this event. Baseline cardiac status was assessed from available clinical records; however, comprehensive baseline cardiac phenotyping was not uniformly available across participating centres. Additionally, biopsy or autopsy myocardial samples, if available, were analysed. We evaluated clinical outcomes: (i) deaths associated with myocarditis, (ii) continuation of ICI, and (iii) recurrence of cardiotoxicity. The cause of death was adjudicated from clinical records.

Clinical specimens obtained from the myocardial biopsy were stained using immunohistochemistry (IHC). Immunohistochemistry was performed for CD3, CD4, CD8, and CD68. Given that the histological appearance varies depending on the sampled region, the area with the highest density of inflammatory cells was selected for analysis. The IHC staining was reviewed by two independent pathologists, who counted the inflammatory cells per high-power field (hpf, 40× lens). The results were expressed as the average per hpf and converted to the average per 1 mm^2^ (1 mm^2^ = 4.21 hpf).^15^

### Statistical analysis

Continuous variables are presented as mean ± standard deviation for normally distributed data, and as median with interquartile ranges (IQR: Q1–Q3) for non-normally distributed data. Categorical variables are presented as numbers (percentages). The Mann–Whitney *U* test or Student’s *t*-test were used to compare continuous variables between the groups. Fisher’s exact test was used to compare the proportions of categorical variables between the groups. A two-sided *P* < 0.05 was considered significant. Statistical analyses were performed using SAS version 9.4 (SAS Institute Inc., Cary, NC, USA) and R software version 4.1.2 (R Foundation for Statistical Computing, Vienna, Austria).

## Results

### Baseline characteristics

We enrolled 90 patients [mean age at diagnosis: 68 years (range: 19–88); 69 men (76.7%)] with ICI-associated cardiac toxicity from 23 hospitals in Japan. The median time from ICI initiation to the diagnosis of cardiac toxicity was 33 days (IQR: 21–148). *Table 1* summarizes the baseline characteristics. The most common primary cancers were non-small cell lung cancer (*n* = 28, 31.1%) and renal cell carcinoma (*n* = 15, 16.7%). The most frequently used ICI was nivolumab (*n* = 49, 54.4%), and 13 patients (14.4%) received combination ICI therapy.

**Table 1:** Baseline characteristics of study participants

	Total *n* = 90	Grade 1 *n* = 37	Grades 2–4 *n* = 53	*P*-value
Age	67.5 ± 13.5	67.4 ± 13.2	67.6 ± 13.8	
Male	69 (76.7)	30 (81.1)	39 (73.6)	0.46
Hypertension	41 (45.6)	19 (51.4)	22 (41.5)	0.40
Diabetes	22 (24.4)	10 (27.0)	12 (22.6)	0.80
Dyslipidaemia	19 (21.1)	3 (8.1)	16 (30.2)	0.017
Smoking	52 (57.8)	23 (62.2)	29 (54.7)	0.52
Chronic kidney disease	13 (14.4)	5 (13.5)	8 (15.1)	1.00
Coronary artery disease	9 (10.0)	1 (2.7)	8 (15.1)	0.076
Previous hospitalization due to HF	6 (6.7)	1 (2.7)	5 (9.4)	0.39
LVEF (%)	66.7 ± 12.9	65.8 ± 6.3	61.1 ± 15.2	0.19
Thymoma	2 (2.2)	0 (0.0)	2 (3.8)	0.51
Metastatic disease	39 (43.3)	15 (40.5)	24 (45.3)	0.67
Pre-ICI medications				
Renin-angiotensin system inhibitor	21 (23.3)	8 (21.6)	13 (24.5)	0.80
Beta-blocker	9 (10.0)	3 (8.1)	6 (11.3)	0.73
Mineralocorticoid receptor antagonist	0 (0.0)	0 (0.0)	0 (0.0)	–
Sodium-glucose cotransporter 2 inhibitor	0 (0.0)	0 (0.0)	0 (0.0)	–
Loop diuretic	6 (6.7)	2 (5.4)	4 (7.5)	1.00
Glucocorticoids	3 (3.3)	3 (8.1)	0 (0.0)	0.066
Immunomodulator	0 (0.0)	0 (0.0)	0 (0.0)	–
Type of cancer				
Non-small cell lung cancer	28 (31.1)	11 (29.7)	17 (32.1)	1.00
Renal cell carcinoma	15 (16.7)	5 (13.5)	10 (18.9)	0.58
Gastric cancer	12 (13.3)	8 (21.6)	4 (7.5)	0.065
Head and neck cancer	12 (13.3)	6 (16.2)	6 (11.3)	0.54
Melanoma	8 (8.9)	2 (5.4)	6 (11.3)	0.46
Urinary tract urothelial cancer	4 (4.4)	1 (2.7)	3 (5.7)	0.64
Others	11 (12.2)	4 (10.8)	7 (13.2)	1.00
Prior chemotherapy or radiation				
Chemotherapy	48 (53.3)	25 (67.6)	23 (43.4)	0.032
Anthracycline	1 (1.1)	0 (0.0)	1 (1.9)	1.00
Thoracic irradiation	15 (16.7)	6 (16.2)	9 (17.0)	1.00
ICI type				
Nivolumab (anti-PD1)	49 (54.4)	24 (64.9)	25 (47.2)	0.13
Combination therapy (nivolumab + ipilimumab)	13 (14.4)	2 (5.4)	11 (20.8)	0.065
Pembrolizumab (anti-PD1)	22 (24.4)	7 (18.9)	15 (28.3)	0.33
Atezolizumab (anti-PDL1)	12 (13.3)	3 (8.1)	9 (17.0)	0.35
Durvalumab (anti-PDL1)	4 (4.4)	1 (2.7)	3 (5.7)	0.64
Avelumab (anti-PDL1)	3 (3.3)	2 (5.4)	1 (1.9)	0.57

Mean ± standard deviation, or *n* (%).

anti-CTL4, anti-cytotoxic T-lymphocyte-associated protein 4; anti-PD1, anti-programmed cell death protein 1; anti-PDL1, anti-programmed death-ligand 1; ICI, immune checkpoint inhibitor; LVEF, left ventricular ejection fraction.

**Figure 1:**
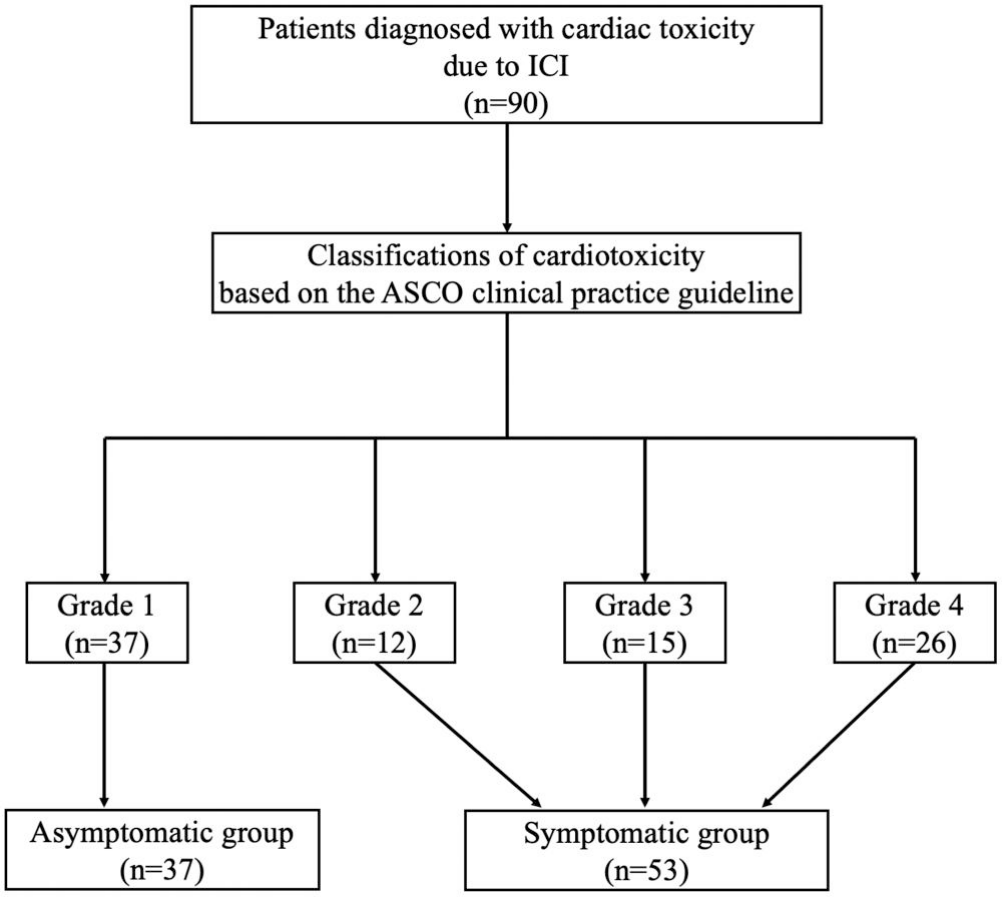
Flow diagram showing the recruitment of patients with cardiac toxicity. We classified cardiac toxicity due to ICI according to the American Society of Clinical Oncology Clinical Practice Guideline, from Grade 1 to 4. ASCO, American Society of Clinical Oncology; ICI, immune checkpoint inhibitor.

With respect to the severity of cardiotoxicity, 37 patients (41.1%) were classified as Grade 1, 12 (13.3%) as Grade 2, 15 (16.7%) as Grade 3, and 26 (28.9%) as Grade 4 (*Figure 1*). *Table 2* shows the baseline characteristics according to these grades. Notably, two patients with thymoma were included in the symptomatic group (Grades 2–4), while three patients who were already taking oral glucocorticoids were categorized in the asymptomatic group (Grade 1). Prior chemotherapy was significantly more common in the asymptomatic group than in the symptomatic group. The proportion of patients with metastatic disease did not differ significantly between asymptomatic and symptomatic groups (40.5% vs. 45.3%, *P* = 0.67).

**Table 2:** Baseline characteristics of study participants by cardiotoxicity-grade

	Total *n* = 90	Grade 1 *n* = 37	Grade 2 *n* = 12	Grade 3 *n* = 15	Grade 4 *n* = 26
Age	67.5 ± 13.5	67.4 ± 13.2	66.3 ± 18.0	70.8 ± 13.1	67.5 ± 13.5
Male	69 (76.7)	30 (81.1)	8 (66.7)	10 (66.7)	21 (80.8)
Hypertension	41 (45.6)	19 (51.4)	3 (25.0)	9 (60.0)	10 (38.5)
Diabetes	22 (24.4)	10 (27.0)	1 (8.3)	4 (26.7)	7 (26.9)
Dyslipidaemia	19 (21.1)	3 (8.1)	3 (25.0)	6 (40.0)	7 (26.9)
Smoking	52 (57.8)	23 (62.2)	5 (41.7)	10 (66.7)	14 (53.8)
Chronic kidney disease	13 (14.4)	5 (13.5)	1 (8.3)	3 (20.0)	4 (15.4)
Coronary artery disease	9 (10.0)	1 (2.7)	2 (16.7)	5 (33.3)	1 (3.8)
Previous hospitalization due to HF	6 (6.7)	1 (2.7)	1 (8.3)	1 (6.7)	3 (11.5)
Thymoma	2 (2.2)	0 (0.0)	1 (8.3)	0 (0.0)	1 (3.8)
Prior chemotherapy or radiation					
Prior chemotherapy	48 (53.3)	25 (67.6)	5 (41.7)	9 (60.0)	9 (34.6)
Anthracycline	1 (1.1)	0 (0.0)	0 (0.0)	0 (0.0)	1 (3.8)
Thoracic irradiation	15 (16.7)	6 (16.2)	3 (25.0)	3 (6.7)	3 (11.5)

Mean ± standard deviation, or *n* (%).

### Findings at cardiotoxicity onset

In the symptomatic group, the median time from the first ICI administration to the onset of cardiac toxicity was 30 days (IQR: 21–125), compared with 35 days (IQR: 14–156) in the asymptomatic group. *Table 3* summarizes the cardiac test findings at the onset of cardiac toxicity. One patient in each group was receiving anti-vascular endothelial growth factor receptor tyrosine kinase inhibitor (VEGFR-TKI) in combination with ICI. At the time of onset, patients in the symptomatic group had significantly higher troponin I and creatine kinase (CK) levels compared with those in the asymptomatic (Grade 1) group. In addition, 40% of symptomatic patients had a left ventricular ejection fraction (LVEF) below 50% on transthoracic echocardiography. Other organ-specific irAEs are presented in *Table 4*. The most common irAE was myositis (12.2%), followed by adrenal insufficiency (7.8%) and myasthenia gravis (6.7%).

**Table 3:** Cardiac findings at the onset of cardiac toxicity

	Total *n* = 90	Grade 1 *n* = 37	Grades 2–4 *n* = 53	*P-*value
Troponin I (pg/mL)	112.2 (23.6–851.8) *n* = 42	42.6 (12.5–77.0)	475.5 (48.9–3001.0)	<0.01
Troponin T (ng/mL)	0.139 (0.048–0.442) *n* = 25	0.139 (0.069–0.156)	0.135 (0.046–0.450)	0.72
BNP (pg/mL)	117 (46–416) *n* = 45	68 (29–254)	225 (66–554)	0.10
NT-proBNP (pg/mL)	600 (171–2091) *n* = 28	266 (164–908)	1110 (556–2816)	0.19
CK (U/L)	257 (70–946) *n* = 87	104 (50–616)	317 (112–1416)	0.012
CK-MB (IU/L)	36.8 (8.0–101.3) *n* = 38	37.5 (24.5–69.0)	33.4 (7.3–114.0)	1.00
CK-MB (ng/mL)	4.0 (1.5–12.1) = 19	2.3 (1.4–2.8)	4.5 (1.6–16.0)	0.13
Rhythm				
Sinus	72 (80.0)	32 (86.5)	40 (75.5)	0.29
Sinus tachycardia	14 (15.6)	3 (8.1)	11 (20.8)	0.14
Sinus bradycardia	2 (2.2)	1 (2.7)	1 (1.9)	1.00
Atrial fibrillation	8 (8.9)	4 (10.8)	4 (7.5)	0.71
Atrial flutter	1 (1.1)	1 (2.7)	0 (0.0)	0.41
Escape rhythm	6 (6.7)	0 (0.0)	6 (11.3)	0.041
Ectopic atrial rhythm	3 (3.3)	0 (0.0)	3 (5.7)	0.27
Echocardiography findings				
LVEF (%)	60.5 (45.5–66.0) *n* = 78	64.0 (56.8–66.4)	57.0 (37.0–66.0)	0.062
LVEF < 50%	23 (25.6)	2 (5.4)	21 (39.6)	<0.01
Interventricular septum thickness (mm)	9.1 (8.1–10.2) *n* = 71	9.1 (8.1–9.9)	9.2 (8.1–10.8)	0.34
Posterior wall thickness (mm)	9.0 (8.3–10.6) *n* = 71	9.0 (8.5–9.7)	9.5 (8.2–11.0)	0.38
Left ventricular diameter at end diastole (mm)	44.2 (41.0–48.4) *n* = 71	43.0 (41.5–48.0)	44.7 (40.8–50.3)	0.38
Pericardial effusion	13 (14.4)	2 (5.4)	11 (20.8)	0.20

Median (Q1–Q3), *n* (%). Upper limit of normal: troponin I 26.2 pg/mL, troponin T 0.014 ng/mL, BNP 18.4 pg/mL, and NT-proBNP 125 pg/mL.

BNP, brain natriuretic peptide; CK, creatine kinase; ICI, immune checkpoint inhibitor; irAE, immune-related adverse events; LVEF, left ventricular ejection fraction; NT-proBNP, N-terminal pro-brain natriuretic peptide.

**Table 4:** Other immune-related adverse events of study participants

	Total *n* = 90	Grade 1 *n* = 37	Grades 2–4 *n* = 53
Myositis	11 (12.2)	2 (5.4)	9 (17.0)
Myasthenia gravis	6 (6.7)	1 (2.7)	5 (9.4)
Adrenal insufficiency	7 (7.8)	2 (5.4)	5 (9.4)
Hypothyroidism	5 (5.6)	1 (2.7)	4 (7.5)
Hyperthyroidism	2 (2.2)	0 (0.0)	2 (3.8)
Hypophysitis	2 (2.2)	0 (0.0)	2 (3.8)
Pneumonitis	4 (4.4)	2 (5.4)	2 (3.8)
Rash	3 (3.3)	2 (5.4)	1 (1.9)
Colitis	3 (3.3)	1 (2.7)	2 (3.8)
Hepatitis	2 (2.2)	0 (0.0)	2 (3.8)
Nephritis	3 (3.3)	2 (5.4)	1 (1.9)
Other	8 (8.9)	0 (0.0)	8 (15.1)

Median (Q1–Q3), *n* (%). Due to the small number of events for each irAE, statistical comparisons between groups were not performed.

irAE, immune-related adverse events

### Certainty of diagnosis for myocarditis

The certainty of ICI-associated myocarditis was evaluated using Bonaca’s criteria.^14^ As shown in *Table 5*, 28 patients were diagnosed with ‘Definite myocarditis,’ 21 with ‘Probable myocarditis,’ and 10 with ‘Possible myocarditis.’ ‘Definite’ and ‘Probable’ myocarditis were more frequent in the symptomatic group, whereas ‘Possible’ myocarditis predominated in the asymptomatic group.

**Table 5:** Certainty of the diagnosis for myocarditis using the Bonaca’s criteria

	Total *n* = 90	Grade 1 *n* = 37	Grades 2–4 *n* = 53
Definite myocarditis	28 (31.1)	2 (5.4)	26 (49.0)
Tissue pathology diagnosis	21 (23.3)	0 (0.0)	21 (39.6)
Probable myocarditis	10 (11.1)	1 (2.7)	9 (17.0)
Possible myocarditis	52 (57.8)	34 (91.9)	18 (34.0)

*n* (%).

### Treatment details for cardiac toxicity and clinical outcomes

*Table 6* shows the management strategies and outcomes for ICI-associated cardiotoxicity varied by symptom status. Use of mechanical circulatory support was significantly more frequent in the symptomatic group than in the asymptomatic group. The proportion of patients receiving glucocorticoid therapy was also significantly higher in the symptomatic group. Additionally, non-steroidal immunosuppressive therapies (mycophenolate mofetil, high-dose immunoglobulin therapy, or plasma exchange) was administered to seven patients.

**Table 6:** Therapeutic interventions for cardiac toxicity-associated immune checkpoint inhibitor therapy and clinical outcomes

	Total *n* = 90	Grade 1 *n* = 37	Grades 2–4 *n* = 53	*P-*value
Mechanical cardiac support	7 (7.7)	0 (0.0)	7 (13.2)	<0.001
Inotropic drug	16 (17.8)	4 (10.8)	12 (22.6)	0.17
CIEDs or temporary pacemaker	7 (7.7)	1 (2.7)	6 (11.3)	0.29
Antiarrhythmic drug	12 (13.3)	4 (10.8)	8 (15.1)	0.76
Renin-angiotensin system inhibitor	14 (15.6)	4 (10.8)	10 (18.9)	0.38
Beta-blocker	18 (20.0)	4 (10.8)	14 (26.4)	0.11
Mineralocorticoid receptor antagonist	10 (11.1)	4 (10.8)	6 (11.3)	1.00
Sodium-glucose cotransporter 2 inhibitor	2 (2.2)	0 (0.0)	2 (3.8)	0.51
Standard-dose glucocorticoids	42 (46.7)	7 (18.9)	35 (66.0)	<0.001
High-dose glucocorticoids	34 (37.8)	5 (13.5)	29 (54.7)	<0.001
Non-steroidal immunosuppressive therapies	7 (7.7)	1 (2.7)	6 (11.3)	0.23
Clinical outcomes				
Death due to myocarditis	7 (7.7)	0 (0.0)	7 (13.2)	0.039
Continuation of ICI	16 (17.8)	10 (27.0)	6 (11.3)	0.091
Recurrence of cardiotoxicity	1 (6.3)	0 (0.0)	1 (16.7)	0.43

*n* (%). High-dose glucocorticoids were defined as methylprednisolone 500–1000 mg/body.

CIEDs, cardiac implantable electronic devices; ICI, immune checkpoint inhibitor; irAE, immune-related adverse event.

Patients in the asymptomatic group were more likely to continue ICI therapy, with only one patient in this group experiencing a recurrence of cardiotoxicity. Supplementary material online, *Table S1* further details the treatment approaches stratified based on cardiotoxicity grade, along with related clinical events.

Notably, seven deaths due to myocarditis occurred in the symptomatic group, whereas cancer-related deaths following cardiotoxicity were observed in 16 patients (43.2%) in the asymptomatic group and in 10 patients (18.9%) in the symptomatic group.

### Pathological findings of the myocardium

**Figure 2:**
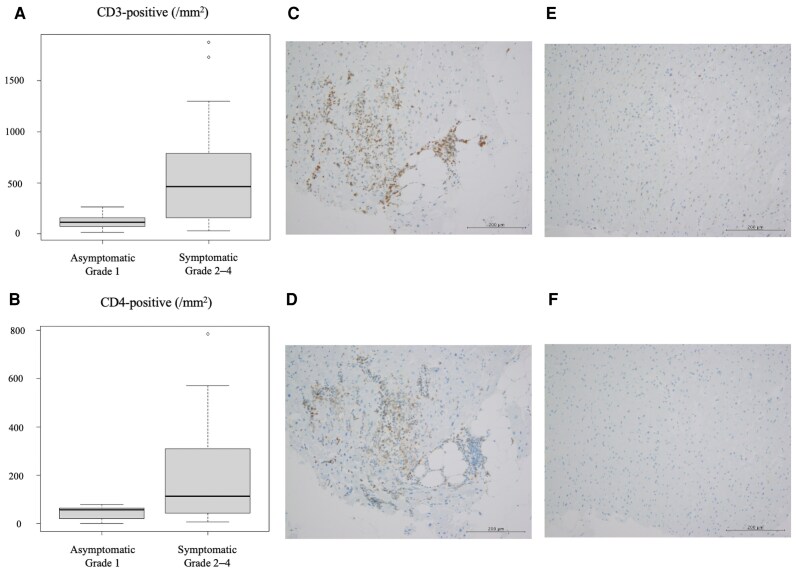
Number of inflammatory cells (CD3 and CD4) infiltrating the myocardium. The number of CD3+ T lymphocytes (*P* = 0.036) (*A*) and CD4+ helper T cells (*P* = 0.059) (*B*) is shown according to the group and severity of cardiac toxicity associated with ICI therapy. Representative immunohistochemistry images from each group of Grades 2–4/Grade 1 (Grades 2–4: *C* and *D*, Grade 1: *E* and *F*). ICI, immune checkpoint inhibitor.

**Figure 3:**
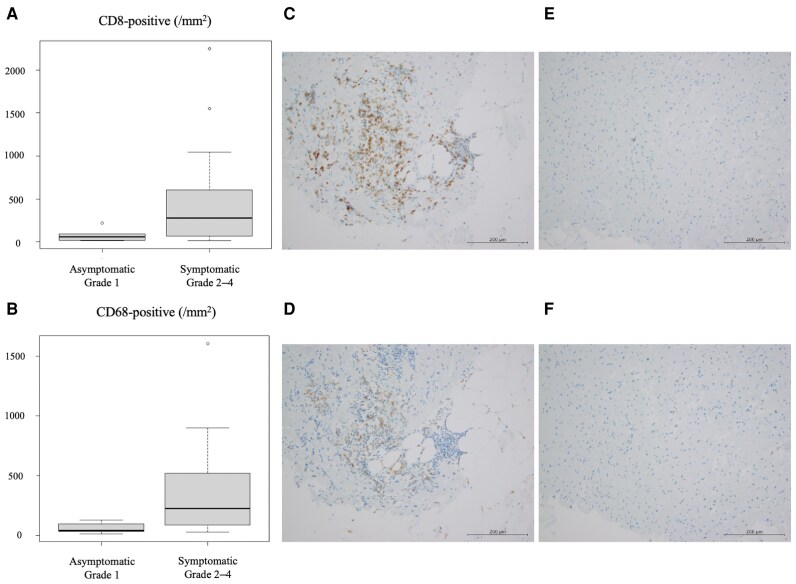
Number of inflammatory cells (CD8 and CD68) infiltrating the myocardium. The number of CD8+ cytotoxic T cells (*P* = 0.064) (*A*) and CD68+ macrophages (*P* = 0.036) (*B*) is shown according to the group and severity of cardiac toxicity associated with ICI therapy. Representative immunohistochemistry images from each group of Grades 2–4/Grade 1 (Grades 2–4: *C* and *D*, Grade 1: *E* and *F*). ICI, immune checkpoint inhibitor.

Myocardial biopsy was performed in 19 symptomatic patients and 5 asymptomatic patients. Supplementary material online, *Table S2* presents the maximum densities of CD3-, CD4-, CD8-, and CD68-positive cells (per mm^2^) in the biopsy samples. Overall, the symptomatic group tended to show a higher infiltration of CD3-, CD8-, and CD68-positive cells than the asymptomatic group (*Figures 2* and *3*). Notably, CD8- and CD68-positive cells increased in number as disease severity progressed (see Supplementary material online, *Figure S1*). In contrast, both groups had relatively few CD4-positive cells infiltrating the myocardium.

## Discussion

This study, the largest of its kind in Japan to focus on ICI-associated cardiac toxicity, provides important insights by comparing asymptomatic and symptomatic cases. Three key findings were identified: (i) in the asymptomatic group, troponin and CK levels and the rate of LVEF decline at the time of cardiac toxicity onset were lower than those in the symptomatic group; (ii) the asymptomatic group had a high rate of ICI continuation with only one case of recurrence observed, whereas seven myocarditis-related deaths occurred in the symptomatic group; and (iii) histopathological samples showed milder inflammation in the asymptomatic group, suggesting a less aggressive disease course. These observations may help inform tailored treatment and follow-up strategies for ICI-associated cardiac toxicity. Recent report also suggests that the severity of cardiomuscular symptoms and magnitude of troponin elevation is associated with the severity of myocarditis.^16^

Our symptomatic cohort broadly resembled the profiles of patients with ICI-associated myocarditis reported in previous studies. Specifically, malignant melanoma, combination ICI therapy, and the use of anti-PD-L1 agents were more common in symptomatic patients—factors already recognized as risk factors or characteristics of ICI-associated myocarditis.^1,7,17–19^ In addition, the symptomatic group showed a higher incidence of myositis (17%), consistent with earlier reports of myositis rates reaching 23–30% in ICI-associated myocarditis.^1,17^ Although the use of VEGFR-TKIs in combination with ICI has been implicated in heightened cardiotoxicity,^20^ only one patient in each group received this therapy. Elevated troponin and CK levels in symptomatic patients in this study align with previous studies suggesting CK as a potentially useful marker associated with severe myocarditis.^21,22^ About half of these patients also demonstrated reduced LVEF, consistent with previous findings.^23^ Using the Bonaca’s criteria, 28 patients were diagnosed as ICI-associated myocarditis, compared with 32 patients diagnosed according to the 2022 European Society of Cardiology Guidelines on cardio-oncology, which represent the current diagnostic standard.^9,14^ Despite aggressive interventions, myocarditis-related deaths were more frequent in the symptomatic group, further suggesting that these patients represent the typical clinical presentation of ICI-associated myocarditis.^14^

The severity of ICI-associated myocarditis varied widely even within the symptomatic population. Compared to the asymptomatic group, the symptomatic group had higher rates of coronary artery disease, dyslipidaemia, and heart failure admissions, indicating that underlying cardiovascular conditions may exacerbate or unmask myocardial damage. Additionally, symptoms may be confounded by non-cardiac irAEs or malignancy itself^24^; hence, even mild myocarditis could present as symptomatic. This may help explain why the mortality rate in the symptomatic group (13.2%) was lower than the previously reported 30–50%.^17,25^ Treatment decisions can be based not only on clinical status (e.g. shock, AV block, ventricular arrhythmias) but also on biopsy findings, as severe inflammation (particularly in Grades 3–4) may warrant intensified immunosuppression, including abatacept or ruxolitinib.^26^ Further analysis of pathological correlates may help refine these treatment pathways.

Recognizing that asymptomatic myocarditis is part of the clinical spectrum is equally important. In certain cases, patients have shown no overt symptoms, yet they were found to have had myocarditis at autopsy.^6^ In this registry, all patients met at least the ‘Possible myocarditis’ level according to Bonaca’s criteria,^14^ and 37 of them were asymptomatic. Their cardiac test abnormalities were mild, and no myocarditis-related deaths occurred—diverging markedly from the 30–50% mortality reported in typical ICI-associated myocarditis.^17,25^ Troponin I is more specific to cardiac muscle than troponin T and may therefore be more appropriate for screening ICI-associated myocarditis. In the present study, serum troponin I levels were significantly elevated in symptomatic group. However, prior studies have also shown that increased troponin alone does not invariably necessitate ICI discontinuation,^7,8,27^ especially as CK levels may be more indicative of significant myocardial injury.^21,22^ Notably, cases with concurrent myocarditis and myositis have been associated with high mortality.^28^ In our cohort, asymptomatic patients had relatively low inflammatory cell infiltration, potentially explaining the favourable outcomes. Conversely, the symptomatic group showed marked elevation in CD8+ and CD68+ cells alongside high troponin and CK levels, reinforcing the link between inflammatory severity and cardiotoxic risk. Early detection and prompt intervention—whether through temporary ICI suspension or glucocorticoid initiation—may also mitigate progression to overt symptoms.

A critical clinical question is whether asymptomatic patients must discontinue ICI therapy. In patients with ICI-associated myocarditis, resumption of ICI therapy was reported to be more common in non-severe cases than in severe cases.^29^ In our study, ∼30% of asymptomatic patients were able to continue ICI without serious cardiac consequences, and this group showed a higher continuation rate than the symptomatic cohort. However, many of these patients died of cancer progression, highlighting the need to balance cardiotoxicity risk with the potential oncologic benefits of continued immunotherapy. Therefore, in cases of asymptomatic myocardial damage, maintaining ICI therapy may be considered in selected patients, provided that patients are closely monitored and timely intervention are promptly initiated if cardiac findings worsen. However, the number of myocardial biopsy samples in the asymptomatic group was limited, which limits the robustness of the pathological comparisons. Therefore, these findings should be interpreted with caution and considered exploratory. Larger studies with more biopsy-confirmed myocarditis cases are required to validate these observations.

### Limitations

This study has some important limitations. First, the retrospective registry design limits causal inference and may introduce bias in the evaluation of diagnostic tests and treatment decisions. Second, because diagnostic evaluations (including myocardial biopsy) and treatment strategies were determined at the discretion of each participating centre, selection and management bias may have influenced both the detection of cardiotoxicity and the observed clinical outcomes. Third, although this study is the largest of its kind in Japan, the overall sample size remains small. This may limit statistical power and restrict the generalizability of the findings; therefore, the results should be interpreted with caution. However, given the low incidence of ICI-associated myocarditis, these data are still of substantial clinical relevance. Fourth, the patient cohort covers the period from 2020 to 2022, when diagnostic and therapeutic approaches to ICI-associated myocarditis were still evolving, potentially limiting the applicability of the findings to current practice. In addition, cardiac magnetic resonance imaging, despite being currently recommended, was performed in only a limited proportion of patients, reflecting clinical practice during the study period and further restricting the transferability of the results to contemporary standards. Therefore, myocardial inflammation may have been underrecognized in some cases. Furthermore, baseline cardiac and oncological phenotyping was not systematically available, including global longitudinal strain assessment, detailed cardiovascular comorbidities, and cancer-related variables such as tumour burden or treatment intent. These limitations may have introduced unmeasured confounding that could influence both the clinical presentation and outcomes of cardiotoxicity. Fifth, long-term follow-up data on cardiac outcomes were limited, which restricts assessment of late cardiac events and long-term prognosis. Finally, because not all patients underwent cardiac biopsy, selection bias must be considered when interpreting the pathological findings, particularly in the asymptomatic group.

## Conclusions

This study is the largest-scale investigation to examine ICI-related cardiotoxicity in Japan, comparing pathological and biomarker characteristics between symptomatic and asymptomatic patient groups. Our findings show that symptomatic (Grades 2–4) patients closely resemble those with classic ICI-associated myocarditis described in earlier reports. In contrast, asymptomatic (Grade 1) patients present milder abnormalities in both biomarkers and inflammatory cell infiltration, suggesting relatively limited myocardial injury in this subgroup. These findings suggest that, in selected asymptomatic patients with mild biomarker elevation and limited inflammatory infiltration, continuation of ICI therapy may be considered under careful cardiac monitoring, rather than routine discontinuation. This distinction may help clinicians balance cardiovascular safety with oncological benefit in the management of ICI-associated cardiac toxicity. Larger prospective studies are warranted to further clarify the clinical significance of asymptomatic cardiac involvement during ICI therapy.

## Supplementary Material

oeag079_Supplementary_Data

## Data Availability

The data underlying this article cannot be shared publicly due to internal regulations and patient consent. Researchers interested in collaboration are invited to contact the corresponding author.
