# Supplementary material for: Asymptomatic and symptomatic cardiac toxicity associated with immune checkpoint inhibitors: insights from a Japanese registry
Source: Eur Heart J Open. 2026 May 15;6(3):oeag079. doi: 10.1093/ehjopen/oeag079 (PMC13252459; doi:10.1093/ehjopen/oeag079)
Supplement: oeag079_Supplementary_Data [file oeag079_supplementary_data.docx]

**Supplementary Materials**

Supplementary Table 1………………………………………………………………Page 2

Supplementary Table 2………………………………………………………………Page 4

Supplementary Figure 1……………………………………………………………..Page 5

**Supplementary Table 1: Therapeutic interventions and clinical outcomes by cardiotoxicity-grade**

|  | **Total**  **N=90** | **Grade 1**  **N=37** | **Grade 2**  **N=12** | **Grade 3**  **N=15** | **Grade 4**  **N=26** |
| --- | --- | --- | --- | --- | --- |
| Mechanical cardiac support | 7 (7.7) | 0 (0.0) | 0 (0.0) | 0 (0.0) | 7 (26.9) |
| Inotropic drug | 16 (17.8) | 4 (10.8) | 0 (0.0) | 0 (0.0) | 12 (46.2) |
| Antiarrhythmic device | 7 (7.7) | 1 (2.7) | 0 (0.0) | 0 (0.0) | 6 (23.1) |
| Antiarrhythmic drug | 12 (13.3) | 4 (10.8) | 1 (8.3) | 1 (6.7) | 6 (23.1) |
| Renin-angiotensin system inhibitor | 14 (15.6) | 4 (10.8) | 0 (0.0) | 2 (13.3) | 8 (30.8) |
| Beta-blocker | 18 (20.0) | 4 (10.8) | 1 (8.3) | 4 (26.7) | 9 (34.6) |
| Mineralocorticoid receptor antagonist | 10 (11.1) | 4 (10.8) | 0 (0.0) | 0 (0.0) | 6 (23.1) |
| Sodium glucose cotransporter 2 inhibitor | 2 (2.2) | 0 (0.0) | 0 (0.0) | 0 (0.0) | 2 (7.7) |
| Steroid | 42 (46.7) | 7 (18.9) | 5 (41.7) | 11 (73.3) | 19 (73.1) |
| High-dose glucocorticoids | 34 (37.8) | 5 (13.5) | 4 (33.3) | 9 (60.0) | 16 (61.5) |
| Immunosuppressive therapy | 7 (7.7) | 1 (2.7) | 0 (0.0) | 4 (26.7) | 2 (7.7) |
| **Clinical outcomes** |  |  |  |  |  |
| Death due to myocarditis | 7 (7.7) | 0 (0.0) | 0 (0.0) | 0 (0.0) | 7 (26.9) |
| Continuation of ICI | 16 (17.8) | 10 (27.0) | 2 (18.2) | 3 (20.0) | 1 (3.8) |
| Recurrence of cardiotoxicity | 1 (6.3) | 0 (0.0) | 0 (0.0) | 1 (33.3) | 0 (0.0) |

n (%).

ICI, Immune checkpoint inhibitor; irAE, Immune-related adverse event

**Supplementary Table 2: Pathological findings of cardiac biopsy in participants**

|  | **Grade 1**  **N=5** | **Grade 2-4**  **N=19** | ***p-*value** |
| --- | --- | --- | --- |
| CD3 (/mm^2^) | 112 (14–264) | 464 (29–1878) | 0.0036 |
| CD4 (/mm^2^) | 57 (0–79) | 114 (7–785) | 0.059 |
| CD8 (/mm^2^) | 57 (14–221) | 278 (14–2249) | 0.064 |
| CD68 (/mm^2^) | 43 (14–129) | 228 (29–1607) | 0.036 |

Median (range)


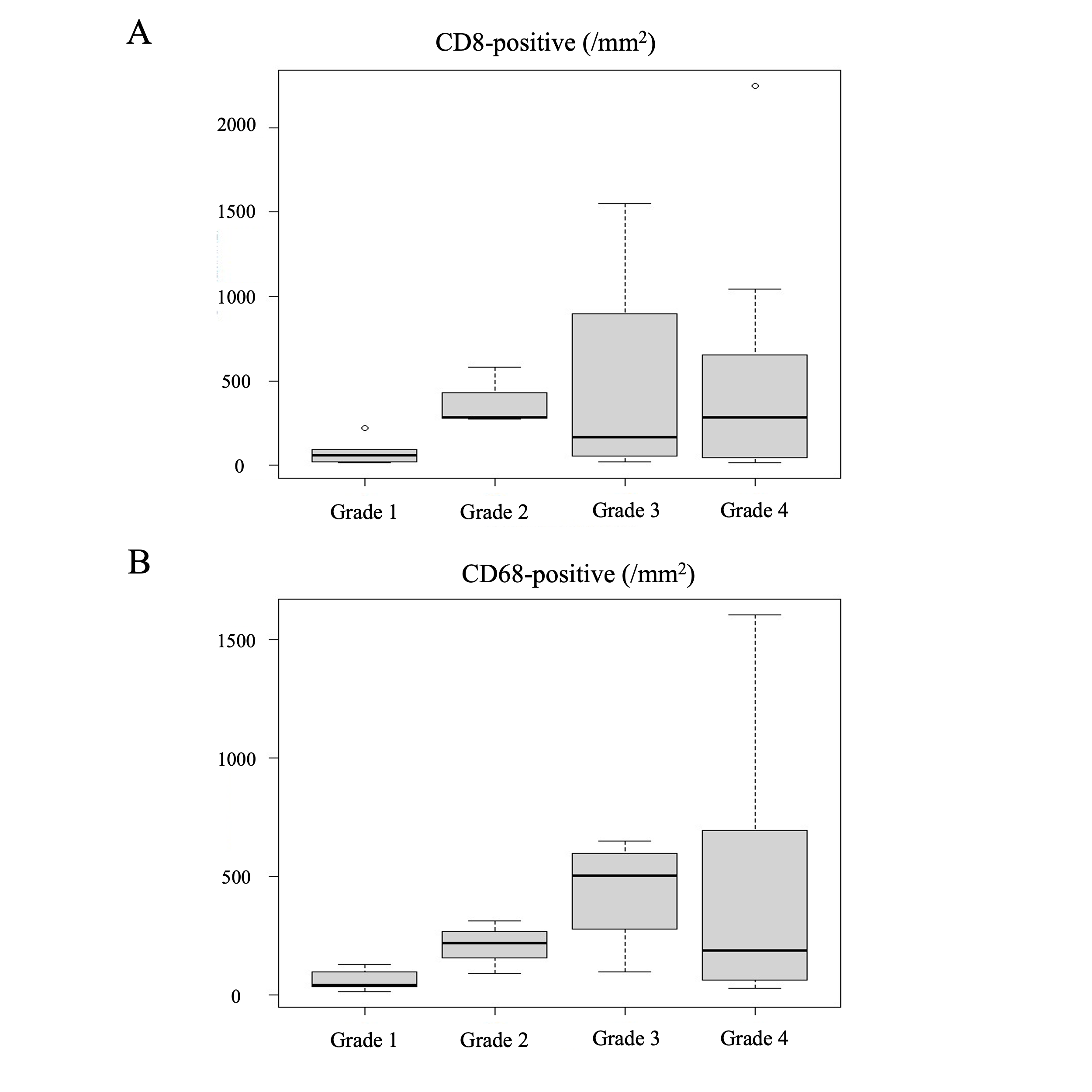


Supplementary Figure 1. Number of inflammatory cells infiltrating the myocardium

The number of CD8-positive cells and CD68-positive cells is shown according to the severity of cardiac toxicity associated with ICI therapy.

ICI, Immune checkpoint inhibitor
